# Appropriateness of quality standards for meaningful intercentre comparisons of aflibercept service provision for neovascular age-related macular degeneration

**DOI:** 10.1038/eye.2017.86

**Published:** 2017-06-23

**Authors:** J S Talks, P James, S Sivaprasad, R L Johnston, M McKibbin, UK Aflibercept Users Group, A J Lotery, F Ghanchi, N Patel, C Bailey, S Mahmood, A Lobo, B Paul, Q Kashif, C Santiago, G Walters, M Tahir, B Mushtaq, k Ahmed, M McKibbin, S Sivaprasad, J Talks

**Affiliations:** 1 Newcastle Eye Centre, Newcastle Upon Tyne Hospitals NHS Foundation Trust, Newcastle Upon Tyne, UK; 2 Institute of Health and Society, Newcastle University, Newcastle Upon Tyne, UK; 3 Kings College Hospital NHS Foundation Trust; NIHR Moorfields Biomedical Research Centre, London, UK; 4 Gloucestershire Hospitals NHS Foundation Trust,London, UK; 5 Leeds Teaching Hospitals NHS Trust, Leeds, UK; 6 Members of the UK Aflibercept Users Group are listed above References.; 7 University of Southampton, Southampton; 8 Bradford Teaching Hospitals Bradford,UK; 9 East Kent Hospitals University NHS Foundation Trust Canterbury, UK; 10 University Hospitals Bristol NHS Foundation Trust, Bristol, UK; 11 Manchester Royal Eye Hospital, Central Manchester University Hospitals NHS Foundation Trust, Manchester, UK;; 12 Moorfields Eye Unit, Bedford Hospital NHS Trust, Bedford, UK;; 13 Barking, Havering and Redbridge University Hospitals NHS Trust, London, UK;; 14 East Sussex Hospitals NHS Trust, Sussex, UK;; 15 Grampian NHS, Aberdeen, UK; 16Harrogate Hospital, Harrogate, UK;; 16 Harrogate Hospital, Harrogate, UK;; 17 Berkshire NHS Trust, Berkshire, UK;; 18 Sandwell and West Birmingham Hospitals NHS Trust, Birmingham, UK;; 19 Aintree NHS Trust, Liverpool, UK;; 20 Leeds Teaching Hospitals NHS Trust, Leeds, UK;; 21 Kings College Hospital NHS Foundation Trust and Moorfields Eye Hospital, London, UK;; 22 Newcastle Upon Tyne Hospitals NHS Foundation Trust, Newcastle upon Tyne, UK.

## Abstract

**Purpose:**

Real-world data give different information on health-care delivery compared with randomised controlled trials. We aimed to evaluate the appropriateness of possible quality standards for intersite comparisons of outcomes of providing Aflibercept for neovascular age-related macular degeneration (nAMD) in clinical practice.

**Patients and methods:**

Retrospective data analysis from an electronic medical record. A consecutive series of treatment-naive patients initiated on aflibercept for nAMD, in the UK from March 2013 to October 2015. Age, visual acuity (VA) at baseline and 1 year, and injection episodes were remotely extracted in an anonymised format.

**Results:**

The mean baseline VA was 54.3 letters, ranging from 51.3 to 58.1 between different centres, in 5620 eyes taken from 12 centres. Out of these, 3360 were initiated on treatment more than a year before. The percentage with <35 letters at baseline was 19.9–3% and that with >70 letters was 24.8–10.7%. Eyes with ≥70 letters at 1 year ranged from 20.2 to 42.9% and those with <35 ranged from 4.5 to 21.6% across different sites. Injection rates in 1 year varied from 5.5 to 8.6, and data available at 1 year also varied from 82.3 to 46.4%.

**Conclusions:**

Significant variation was found between sites attempting to provide the same therapeutic regime. For fair comparisons between sites, we recommend that both VA measures and process measures, such as injection numbers, retention rates, and discharge policies, are used. More work is required to explain the differences. Such real-world data are not generated in the same way as a randomised clinical trial, and maybe best used to help improve service provision.

## Introduction

Anti-vascular endothelial growth factor therapy has been shown to be effective in randomised controlled clinical trials (RCTs) and is the mainstay of treatment for neovascular age-related macular degeneration (nAMD).^1, 2, 3^

Increasingly, real-world data are being used to generate real-world evidence as a way of assessing the performance of a therapy in clinical practice.^4, 5, 6, 7, 8, 9, 10, 11, 12^ RCTs demonstrate the efficacy of an intervention, whereas real-world evidence explores its effectiveness—in other words, how it works in real-world conditions.^13, 14^ A broader range of patients will be treated outside RCTs, and factors such as clinician's decisions, service demands, and differences in assessment methods will influence the results. In ophthalmology, in addition to visual acuity (VA) measures, outcomes such as number of injections and patient compliance with treatment may have important service provision implications. Payers, service providers, and users are becoming more interested in such outcome measures.^15^ Therefore, there is a trend to set benchmarks or quality standards based on large real-world data sets.^4, 5, 6, 7, 8, 9, 10, 11, 12^ However, for data to be used in this way, it is important to establish what outcome measures would be a fair reflection on the quality of a service and the minimum data sets required at each site to allow such comparisons.

In our previous paper, we confirmed that the mean VA improvement is inversely related to starting VA (the worse the baseline VA the greater the gain) and that second presenting eyes often have better baseline VA and maintain better VA than first eyes but have less mean improvement.^11^ We concluded that potentially useful quality standards could be based on the mean presenting VA as a measure of the quality of the referral to treatment pathway, and the percentage of treated eyes with 70 or more VA letters at annual time points as a measure of quality of the clinical care pathway within the service provision.^11^

With real-world data, often VA is recorded with habitual correction rather than full correction, and certainly the recording of VA is not done as robustly as in a clinical trial, which is likely to underestimate the VA.^16^ Large data sets from the United Kingdom should provide a fairer measure of expected VA outcomes for real-life UK practice rather than RCT data. The objective of this study was, therefore, to provide such data and to evaluate a more comprehensive set of outcome measures across multiple sites in the United Kingdom to try and derive quality standards that may be used to evaluate service provision for nAMD and suggest sample sizes that could be used for a fair comparison. We explored whether there were any significant differences in baseline VA, age, number of injections given over a year, percentage of patients remaining under follow-up, and eyes with ≥70 letters at 1 year for treatment-naive patients. We also looked at factors that might influence these measures. For baseline and 1-year mean visual acuities, we looked at the proportion of patients with <35 letters and ≥70 letters at baseline and 1 year in keeping with our proposed quality standards in our previous publication. These standards reflect how patients presented in different centres and the quality of the clinical care pathway. The differences between sites may also represent differences in clinician practice in terms of criteria used to initiate, maintain, and stop treatment.

Data from 16 centres in the United Kingdom working in the same health system that used the same electronic medical record (EMR) to record all clinical data throughout the anti-VEGF care pathway were included. All centres planned to use only aflibercept to treat treatment-naive nAMD, with the intention of following the VIEW protocol.

This is not a RCT but a collection of data from several centres attempting to provide the same treatment in clinical practice. Such data are being used to evaluate service provision; therefore, it is important to explore whether there are significant differences between sites in terms of the proposed quality standards in order to ascertain whether these quality standards are generalisable to be used as fair measures of outcome in the real world.

## Materials and methods

Data were collected to compare the mean baseline VA between sites; the proportion of eyes with <35 letters and >70 letters at baseline; the proportion of first to second affected eyes; the proportion of patients who had the potential to have data at 1 year who had data recorded; the mean number of injections given over 1 year and the proportion with VA≥70 letters at 1 year.

Anonymised data were extracted from 16 United Kingdom National Health Service Hospitals, as detailed in the acknowledgements section, that confirmed they intended to use aflibercept on all treatment-naive eyes with nAMD following the VIEW protocol; three injections 1 month apart and then two monthly for the first year.^1^ The first treatment was initiated in March 2013 and data cutoff for this analysis was October 2015. All data were recorded using a single EMR system (Medisoft Ophthalmology, Medisoft Limited, Leeds, UK), which mandated collection of a standardised data set throughout the nAMD care pathway, which included VA, and injections given at each visit. The lead clinician and Caldicott Guardian (responsible nominee for data protection) at each NHS Hospital gave written approval for anonymised data extraction. Anonymized database analyses of this type do not require ethical permission as they are viewed as audit or service evaluation (see http://www.hra.nhs.uk/research-community/beforeyou-apply/determine-whether-your-study-is-research/). This study was conducted in accordance with the declaration of Helsinki and the UK Data Protection Act.

Although this study is retrospective in nature, the data set mandated by the EMR was defined prospectively before first data entry and hence the study methodology is somewhat closer to an electronic case report form used in clinical trials than a conventional analysis of unstructured data in a retrospective chart review.

### VA imputation

ETDRS (Early treatment diabetic retinopathy study) VA letter scores were recorded at 2 m at each visit at all sites. At each visit, the best-measured VA value was used in analysis. Most VA values were recorded using habitual correction rather than with refraction. Values corresponding to count fingers, hand movements, perception of light (PL) and no PL were substituted with values of 0 letters. In order to be able to plot VA at monthly time points and to maximise the sample size of eyes contributing to the data at each time point, a limited form of data interpolation was used such that a missing month(s) VA value was interpolated based on the mean of the VA letter score before and after the missing time point.

### Statistical methods

Continuous normally distributed data were summarised using means, SEMs, and quartiles. Skewed continuous data were summarised using the medians and quartiles. Despite the skewness and kurtosis of the VA letter score data, the large sample sizes allowed parametric methods to be used for comparison of the means of groups. To enable this, and to include a site in the full analysis, the site had to have a sample size of >30 at 1 year. *T*-tests were used to compare the means of two groups, and analysis of variance was used to compare more than two groups.

Logistic regression was used to model the dependence of the proportion with ≥70 VA letter score on both continuous and categorical predictors, and odds ratios with 95% CIs were reported. All *P*-values were two-sided and statistical significance was taken as *P*<0.05 throughout the analyses. A Bonferroni adjustment was used to guard against inflation of Type I error due to multiple testing.

## Results

The 16 sites had recorded data on 5815 treatment-naive eyes receiving aflibercept for nAMD at the time of data cutoff. Four sites were excluded from further analysis because of low numbers expected to have reached 52 weeks due to delays in them starting using aflibercept for treatment-naive nAMD. The mean age of the patients was 80.0 years (median 81.0 years) and 63.5% were women. Data for comparison of VA at baseline were available on 5620 eyes from 12 sites (A–P). At 1 year data were available on 2412 (71.8%) eyes from a possible 3360.

The number of eyes varied per site from 177 to 1138. Table 1 shows the mean baseline VA scores and SEs. The mean baseline VA between sites varied from 51.3 to 58.1 letters, with a median from 53 to 60 letters. For the mean, sites A and G had significantly higher scores, while sites D, F, and L had significantly lower scores.

With a sample size of 198 per site, we were able to detect with 80% statistical power a difference of four letters from a target value of 54, this being the mean VA. This assumes that Type I error is 5% (this is the probability that one can falsely conclude that any of 12 sites has baseline score significantly different from 54). With a sample size of 352, we can similarly detect a difference of three letters from the target value.

At baseline, 637 (11.3%) out of 5620 eyes had poor VA (≤35 letters; Table 2). The proportion with <35 letters varied by site, with significantly higher percentages of 17.5%, 19.9%, and 16.3%, respectively, for sites D, F, and L, and significantly lower percentages of 7.7% and 3.0%, respectively, for sites C and G.

In all, 985 (17.5%) out of the 5620 eyes had good VA (≥70 letters) at baseline. This proportion also varied by site with significantly higher percentages of 24.8% and 22.5%, respectively, for sites A and G and significantly lower percentages of 11.7%, 10.7%, and 13.2%, respectively, for sites D, F, and L.

In all, 1200 (21.4%) out of the 5620 eyes treated at baseline were second eyes. There was little variation by site and the differences were not statistically significant.

In all, 2412 (71.8%) out of 3360 eyes initiated on aflibercept 1 year or more from initial treatment were still being followed up at 1 year from baseline (Table 3). This proportion also varied by site, with significantly higher proportion of 82.3% and 78.2%, respectively, for sites A and G, and significantly lower percentages of 58.4% and 46.4%, respectively, for sites B and K.

The median number of injections recorded was 7 with an interquartile range of 3.

Sites D, F, and G gave a significantly higher number of injections of, respectively, 8.2, 8.6, and 8.1, while sites K and L gave a significantly lower numbers of injections of, respectively, 5.5 and 5.8.

With a sample size of 194 in a site we were able to detect with 80% statistical power a difference of 5% from a target value of 25% for having ≥70 letters at 1 year. This assumes that Type I error is 5% (this is the probability that one can falsely conclude that any of 12 sites has percentage different from 25). With a sample size of 302, we can similarly detect a difference of 4% from the target value.

The 2412 eyes with letter scores at 52 weeks were analysed, and it was found that the percentage of eyes achieving >70 letters at 52 weeks varied between 20.2% for site N to 42.8% for site G, while overall the percentage was 33.4% (Table 4). The proportion of patients under follow-up with <35 letters at 1 year varied from 4.5 to 21.6%

A logistic regression model was fitted to the data, and this showed that the prevalence of eyes achieving ≥70 letters at 52 weeks increases in proportion to the number of injections: an increase of one injection increases the odds of ≥70 letters by 4% (odds ratio is 1.04 with 95% CI 1.01–1.08; Table 5). The prevalence of this outcome decreases with age: those aged 70–79, 80–84, and 85+ have reduced prevalence with odds ratios of 0.68, 0.49, and 0.37, respectively. This prevalence also increases with baseline VA letter scores. Relative to subjects with letter scores of 50–59 at baseline, a baseline score of under 45 letters decreases the odds of ≥70 VA letter score by 76% (odds ratio of 0.24 with 95% CI 0.17–0.35). Conversely, a baseline score of 60–64 increases the odds by 57% (odds ratio is 1.47 with 95% CI 1.16–2.13). Attending site G increased the odds by 53% (odds ratio with 95% CI 1.15–2.05). Possible interactions between age and number of injections were tested for and found not to be significant.

## Discussion

In our previous publication on 1840 treatment-naive eyes on aflibercept therapy for nAMD, we found that the amount of VA gain depended on the presenting VA and on whether a first or second affected eye was being treated as second affected eyes are often initiated on treatment with better baseline VA.^11^ Therefore, a change in the mean VA should not be used as the only indicator of quality of care. We also suggested that a good measure of the benefit of a treatment and the quality of the service provision is defined as the proportion of patients who achieve ≥70 letters at a measured time point as this VA outcome is equivalent to driving standard in the United Kingdom.

With a larger sample size in this study, we have been able to make meaningful statistical comparisons between the sites and observed significant differences between sites for both mean baseline VA and the proportion of eyes with ≥70 letters at 1 year. A minimum number of consecutive patients’ data at a site is required to make meaningful intersite comparisons. This study showed that with a sample size of 198 in a site we were able to detect with 80% statistical power a difference of four letters from a proposed target value of baseline VA of 54 letters, and with a sample size of 194 we were able to detect with 80% statistical power a difference of 5% from a target value of 25% for the number of eyes with ≥70 letters at 1 year.

Eyes starting with ≤35 letters varied from 19.9 to only 3%, and eyes starting with ≥70 letters ranged from 10.7 to 24.8%. Therefore, based on our study results, we recommend the use of multiple outcome measures in evaluating services as each outcome measure is confounded by non-service-related factors or inherent bias. For example, the mean starting VA is influenced by many factors including awareness of macular degeneration in the community; access to eye services particular community optometry or emergency eye care; referral pathways for patients with suspect nAMD; and promptness with which treatment is actually started once suspected or diagnosed. Delay at any stage on this pathway leads to a lower baseline VA.^17, 18^ Improvements in every step of the nAMD care pathway are likely to lead to better baseline VAs, which are the biggest determinant of VA at 1 year. In addition, the mean baseline VA measurement may also be influenced by first/second eye differences or differences in opinion between clinicians on the level of VA, both good and bad, for which they would start treatment.

Second affected eyes tend to have higher baseline VA, usually because these eyes are under regular surveillance after initiating treatment in the first eyes. Not all patients with nAMD will be symptomatic at ≥70 letters and be aware of the urgency of seeking help. The proportion of first to second eyes could in theory influence the mean baseline score at each site, but in reality there were only small differences between sites.

A confounding factor, if real-world data are mainly being used to compare to RCTs, is the method of VA measurement, as in many real-world data collections, VA is measured with habitual correction rather than full refraction. It is therefore likely that VA measurements maybe underestimated.

In order to enable fair comparisons between sites, we recommend that multiple measures are included that assess both VA measures and process measures such as injection numbers, retention rates, and discharge policies.

Our logistic regression model found that the odds of eyes having ≥70 letters at 1 year were best with a higher baseline VA, younger age of patient, higher number of injections, and by attending site G. In this study, sites had intended to follow the VIEW protocol, but there was a difference in the mean numbers of injections given. Fewer injections are frequently associated with worse VA outcomes in many studies.^19^ Assessing a service on this parameter alone is insufficient as the number of injections given at a site depend on multiple factors, for example, the ability of the health-care service to provide the appropriate appointment intervals, patient attendance, and success, but also the futility criteria used at each site and whether such patients are followed up while not being treated or discharged. This is clearly illustrated with the proportion of patients with <35 letters still under follow-up at 1 year as that varied from 4.5 to 21.6%.

Other confounding factors that determine the outcome measures highlighted in this study include the highly significant differences in the proportions of patients with data at 1 year that varied from 46.4 to 82.3%. This proportion may not mean that the patients are no longer being treated. Patients may move to hospitals especially in larger cities. Therefore, failing to maintain this quality standard may not always reflect the inability of sites to provide timely appointments. Further scrutiny into local factors has to be investigated before assumptions are made on the quality of the service based on this quality standard.

A particular area of concern of benchmarking services based on the set quality standards is that there are several areas in the service provision in nAMD that lack evidence. For example, the clinical benefit and cost effectiveness of initiating treatment in eyes with very poor baseline VA, or maintaining treatment if little improvement is achieved in the first few months, particularly if VA in the fellow eye is good, has not been adequately studied. In cash-limited publicly funded health-care systems such evidence and guidance would be useful as clinicians negotiate with health-care purchasers. Other measures of benefit from treatment would involve quality of life measures such as recommended by the International Consortium for health-care outcomes, macular degeneration data collection guide (http://www.ichom.org/medical-conditions/macular-degenartion/).^20^ These are certainly to be recommended, but in practice can be difficult to carry out in busy clinical practice.

A report on intercentre variation in the United Kingdom from an older data set looking at the PRN use of ranibizumab for AMD also reported variations in service provision. It might be expected that our intersite comparisons would show more similarities between centres as there is now more experience with the use of anti-VEGF in centres and we were all attempting to provide the same regime of fixed dosing resulting in less variations. The study showed that a younger age, better starting VA, and a higher number of injections were associated with better VA outcomes, but we found that significant variation between centres persisted even after adjusting for these factors.^21^ We have highlighted other reasons for these differences.

In conclusion, we have proposed a number of outcomes and sample sizes that could be used together to evaluate the quality of a service. It is apparent that, while the differences we found could represent differences in the quality of care of patients they could also reflect differences in population characteristics as well as difficulties in recording standardised comparable VA measures and differences in a clinical approach, such as being more or less willing to start patients on therapy with lower VA levels and continuing patients under follow-up with lower VA. Differences in retention of patients under follow-up, or at least recording the data on the EMR used, may also be a factor. Other comparator measures could be non-VA based such as the incidence of new patients being treated; time to first treatment and number of visits. Further work is required to define and evaluate a set of appropriate measures to assess quality of care using anti-VEGF in nAMD. However, we recommend that both VA and process review should be evaluated while assessing anti-VEGF clinical care pathways for neovascular AMD. As a minimum we recommend the following to be reported while auditing services: (1) proportion of patients with presenting VA<35 and ≥70 letters, (2) proportion of patients with VA<35 and ≥70 letters at 1 year, (3) number of injections in 1 year, and (4) rate of retention of patients at 1 year. More work will need to be done to recommend the minimum achievable standards. In addition, further analysis of real-world outcomes over multiple years of follow-up will provide us with the information required for on-going yearly service evaluation.


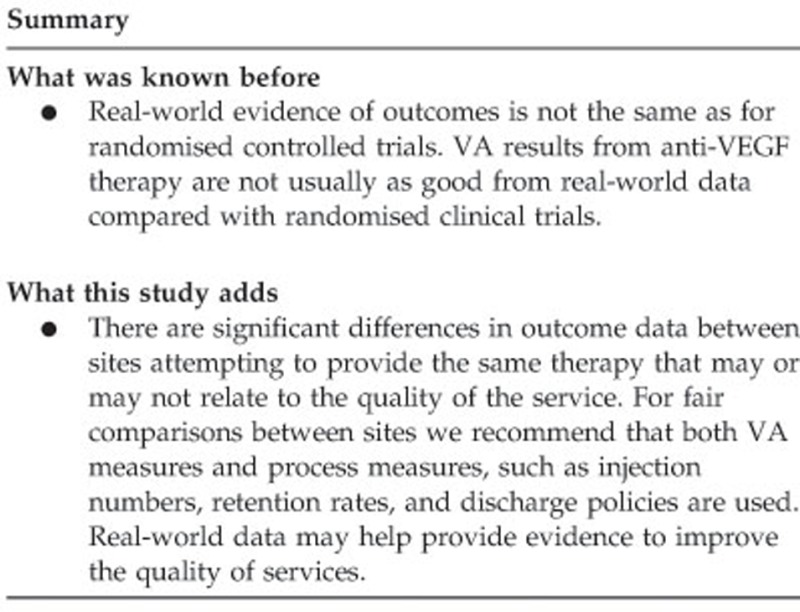


## Figures and Tables

**Table 1:** Baseline visual acuities for eyes by site

*Site*	*Eyes*		*Baseline; median*	*Score; mean*	P-*value*^a^
	N	*%*			
A	1138	20.3	59 (46, 69)	56.6 (0.5)	<0.001^a^
B	483	8.6	58 (45, 67)	55.3 (0.7)	0.151
C	584	10.4	55 (45, 65)	53.8 (0.6)	0.404
D	514	9.2	54 (40, 64)	52.0 (0.7)	0.001^a^
F	271	4.8	55 (39, 65)	51.3 (1.0)	0.003^a^
G	600	10.7	60 (50, 68)	58.1 (0.5)	<0.001^a^
H	329	5.9	55 (42, 65)	53.1 (0.9)	0.165
K	397	7.1	54 (42, 65)	52.1 (0.8)	0.006
L	698	12.4	53 (40, 65)	51.7 (0.6)	<0.001^a^
M	177	3.2	59 (41, 68)	53.5 (1.2)	0.514
N	251	4.5	57 (44, 65)	54.1 (1.0)	0.844
P	178	3.2	59 (42, 68)	55.2 (1.2)	0.467
Total	5620	100.0	57 (44, 66)	54.3 (0.2)	

E, I, J, and O sites were excluded because of having insignificant numbers of patients expected at 1 year, see Materials and methods.

a Significant after Bonferroni adjustment for multiple testing.

**Table 2:** Baseline letter scores of ≤35 and ≥70 by site

*Site*	*Total*	*Letter score*≤*35*			≥*70 Letter score*		
		N	*Percent with 95% CI*	P-*value*^a^	N	*Percent with 95% CI*	P*-value*^a^
A	1138	103	9.1 (6.6, 11.5)	0.007	282	24.8 (21.1, 28.4)	<0.001^b^
B	483	43	8.9 (5.2, 12.6)	0.061	94	19.5 (14.3, 24.6)	0.283
C	584	45	7.7 (4.5, 10.9)	0.001^b^	78	13.4 (9.3, 17.4)	0.003^b^
D	514	90	17.5 (12.7, 22.3)	<0.001^b^	60	11.7 (7.6, 15.7)	<0.001^b^
F	271	54	19.9 (13.0, 26.9)	<0.001^b^	29	10.7 (5.3, 16.1)	<0.001^b^
G	600	18	3.0 (1.0, 5.0)	<0.001^b^	135	22.5 (17.6, 27.4)	0.004^b^
H	329	49	14.9 (9.3, 20.5)	0.070	53	16.1 (10.3, 21.9)	0.484
K	397	62	15.6 (10.4, 20.8)	0.019	54	13.6 (8.7, 18.5)	0.023
L	698	114	16.3 (12.3, 20.3)	<0.001^b^	92	13.2 (9.5, 16.8)	0.001^b^
M	177	19	10.7 (4.1, 17.4)	0.796	36	20.3 (11.7, 29.0)	0.353
N	251	19	7.6 (2.8, 12.4)	0.024	33	13.1 (7.0, 19.3)	0.040
P	178	21	11.8 (4.9, 18.7)	0.848	39	21.9 (13.0, 30.8)	0.157
Total	5620	637	11.3 (10.1, 12.5)		985	17.5 (16.1, 19.0)	

a *P*-value for comparison with percentage for all sites.

b Significant after Bonferroni adjustment for multiple testing.

**Table 3:** Percentage of eyes still being treated 1 year after baseline by site

*Site*	*Still treated?*		*% treated*		
	*No*	*Yes*	*Total*	*With 95% CI*	P*-value*^a^
A	132	613	745	82.3 (78.3, 86.3)	<0.001^b^
B	99	139	238	58.4 (49.2, 67.6)	<0.001^b^
C	122	241	363	66.4 (59.3, 73.5)	0.030
D	67	223	290	76.9 (69.8, 84.0)	0.039
F	42	115	157	73.2 (63.1, 83.4)	0.679
G	81	290	371	78.2 (72.0, 84.3)	0.003^b^
H	44	115	159	72.3 (62.2, 82.5)	0.879
K	155	134	289	46.4 (38.0, 54.8)	<0.001^b^
L	127	299	426	70.2 (63.8, 76.5)	0.471
M	37	74	111	66.7 (53.8, 79.5)	0.253
N	24	94	118	79.7 (69.0, 90.3)	0.034
P	18	75	93	80.6 (68.9, 92.4)	0.031
Total	948	2412	3360	71.8 (69.6, 74.0)	

a *P*-value for comparison with percentage for all sites.

b Significant after Bonferroni adjustment for multiple testing.

**Table 4:** Eyes with ≤35 and ≥70 VA letter score at 52 weeks by site

		*≤35 VA letter score*			≥*70 VA letter score*		
*Site*	*total*	*N*	*Percent with 95% CI*	P-*value*^a^	N	*Percent with 95% CI*	P*-value*^a^
A	613	55	9.0 (5.7, 12.3)	0.015	228	37.2 (31.6, 42.8)	0.050
B	139	10	7.2 (0.9, 13.5)	0.037	56	40.3 (28.4, 52.2)	0.097
C	241	28	11.6 (5.7, 17.5)	0.940	80	33.2 (24.5, 41.9)	0.953
D	223	42	18.8 (11.3, 26.3)	0.007	69	30.9 (22.1, 39.8)	0.432
F	115	20	17.4 (7.3, 27.5)	0.112	32	27.8 (15.9, 39.8)	0.184
G	290	13	4.5 (1.0, 8.0)	<0.001^b^	124	42.8 (34.4, 51.1)	0.001^b^
H	115	15	13.0 (4.0, 22.0)	0.686	34	29.6 (17.4, 41.8)	0.371
K	134	21	15.7 (6.7, 24.7)	0.215	36	26.9 (15.9, 37.8)	0.089
L	299	43	14.4 (8.6, 20.2)	0.199	81	27.1 (19.7, 34.5)	0.014
M	74	16	21.6 (7.9, 35.3)	0.040	18	24.3 (10.0, 38.6)	0.070
N	94	13	13.8 (3.6, 24.0)	0.564	19	20.2 (8.3, 32.1)	0.001^b^
P	75	8	10.7 (0.5, 20.9)	0.756	28	37.3 (21.3, 53.3)	0.478
Total	2412	284	11.8 (9.9, 13.7)		805	33.4 (30.6, 36.1)	

a *P*-value for comparison with percentage for all sites.

b Significant after Bonferroni adjustment for multiple testing.

**Table 5:** Influence of factors on VA letter score of 70 letters or more at 52 weeks

*Factor*	*Odds ratio*	P*-value*
Number of injections	1.04 (1.01, 1.08)	0.007
*Age at baseline*^a^		
70–79	0.68 (0.49, 0.93)	0.018
80–84	0.49 (0.35, 0.69)	<0.001
85 and over	0.37 (0.26, 0.52)	<0.001

*Baseline visual acuity letters*^b^		
Under 45	0.24 (0.17, 0.35)	<0.001
45–49	0.46 (0.31, 0.71)	<0.001
60–64	1.57 (1.16, 2.13)	<0.001
65–69	3.32 (2.47, 4.47)	<0.001
70–74	5.65 (4.08, 7.83)	<0.001
75+	10.52 (6.75, 16.39)	<0.001

Site G	1.53 (1.15, 2.05)	<0.001

a Reference is aged 0–69 years.

b Reference is 50–59 ETDRS letters.
